# Tight controlled dose reduction of biologics in psoriasis patients with low disease activity: a randomized pragmatic non-inferiority trial

**DOI:** 10.1186/s12895-017-0057-6

**Published:** 2017-05-08

**Authors:** Selma Atalay, Juul M. P. A. van den Reek, Lieke J. van Vugt, Marisol E. Otero, Peter C. M. van de Kerkhof, Alfons A. den Broeder, Wietske Kievit, Elke M. G. J. de Jong

**Affiliations:** 1 Department of Dermatology, Radboud University Medical Center, Rene Descartesdreef 1, 6525 GL Nijmegen, The Netherlands; 2 Department of Rheumatology, Sint Maartenskliniek, Hengstdal 3, 6574 NA Ubbergen, Nijmegen, The Netherlands; 3 Radboud University, Comeniuslaan 4, 6525 HP Nijmegen, The Netherlands; 4 Department for Health Evidence, Radboud University, Geert Grooteplein 21, 6525 EZ Nijmegen, The Netherlands

**Keywords:** Psoriasis, Dose reduction, Biologics, Non- inferiority, Adalimumab, Etanercept, Ustekinumab, Therapy, Cost-effectiveness

## Abstract

**Background:**

Psoriasis is an immune-mediated chronic inflammatory skin disorder for which several targeted biologic therapies became available in the last 10 years. Data from patients with rheumatoid arthritis revealed that dose tapering combined with tight control of disease activity is successful. For psoriasis patients the lowest effective dose of biologics needs to be determined.

The objective was to assess whether dose tapering of biologics guided by Psoriasis Area and Severity Index (PASI) and Dermatology Quality of Life Index (DLQI) scores in psoriasis patients with controlled disease activity is non-inferior (NI) to usual care.

**Methods/design:**

This is a multicenter, pragmatic, randomized, non-inferiority trial with cost- effectiveness analysis. One hundred and twenty patients with stable low disease activity (PASI ≤ 5 and DLQI ≤ 5) for at least 6 months with a stable use of adalimumab, etanercept or ustekinumab will be randomized 1:1 to the dose reduction group or usual care. In the dose reduction group, the treatment intervals will be prolonged stepwise, resulting in a 33% and 50% dose reduction, respectively. Disease activity is monitored every three months with PASI and DLQI. In case of flare the treatment is adjusted to the previous effective dose. The primary outcome (PASI) at 12 months will be analyzed with ANCOVA in which the baseline PASI will be included as covariate to gain efficiency.

The secondary outcomes include number of and time to disease flares, health-related quality of life, serious adverse events, and costs.

**Discussion:**

With this study we want to assess whether disease activity guided dose reduction of biologics can be achieved for psoriasis patients with low stable disease activity, without losing disease control. By using the lowest effective dose of biologics, we expect to minimize side effects and save costs.

**Trial registration:**

This trial was registered at ClinicalTrials.gov (NCT 02602925). Trial registration date October 9 2015.

## Background

Psoriasis is an immune-mediated chronic inflammatory skin disorder, affecting 2–3% of the world population. It is characterized by erythematous scaly plaques and associated with several significant comorbidities such as psoriatic arthritis. Patients with moderate to severe psoriasis have a high disease burden, the impairment of disease-related quality of life is comparable to that of patients with cancer and depression [1].

Several targeted biologic therapies have become available for psoriasis patients such as TNF-alpha inhibitors (etanercept, adalimumab) and anti-IL-12/IL- 23 agents (ustekinumab). These drugs block critical cytokine pathways implicated in the pathophysiology of psoriasis. Multiple trials have been conducted to study the safety and efficacy of etanercept, adalimumab and ustekinumab [2–4]. These biologics are widely used in daily practice.

Although, biologics are considered as relatively safe, side effects do still occur, mainly due to immunosuppressive effects. Especially in patients with chronic inflammatory diseases such as psoriasis, where lifelong treatment is considered necessary to achieve disease control, it is important to minimize the chance of side effects. In addition, biologic treatment is expensive and imposes a high burden on the national health care expenditures [4, 5]. Lowering the overall exposure to biologics could result in both a lower risk of side effects and substantial health care savings. We know from small studies that withdrawal of the biologic showed quick disease recurrence in 99% of patients with psoriasis [2, 3, 6–11]. Moreover, retreatment with the biologic did not always reach the same effectiveness as the first episode of treatment [8–10, 12]. Another option would be in patients with controlled disease to lower the dosage of biologics.

For psoriasis patients, there is lack of evidence of dose-tapering of biologics and the lowest effective dose of biologics in daily practice in the individual patient remains to be determined. There is one poster reporting successful dose tapering in 10 patients using adalimumab and a small retrospective case series in which etanercept was tapered has been published [13, 14]. Another cross-sectional study described the retreatment after tapering of etanercept, which was effective and well tolerated in psoriasis patients. However, this study did not describe what the effect of dose-tapering of etanercept was [9].

Recently, a disease activity guided dose tapering and stopping strategy has been shown to be non- inferior to treatment continuation in patients with rheumatoid arthritis (RA) using adalimumab or etanercept [15]. However, this evidence cannot directly be applied to patients with psoriasis. Psoriasis is different disease with different genetic background and different cytokines in the inflammatory pathways are involved. This indicates differences regarding drug related effectiveness, side-effects and behavioral factors. For example, RA patients are treated in combination with other disease-modifying anti rheumatic drugs, which may influence the disease activity while dose-tapering of the biologic. In psoriasis other biologics are used than in RA and the biologic is mostly prescribed as monotherapy. The biologic can also be combined with other co-medication than in patients with RA.

In this study, we will focus on biologic dose-tapering instead of withdrawal of the biologic. In daily practice, the most widely used biologic treatments are adalimumab, etanercept and ustekinumab.

Therefore, we designed a multicenter, randomized trial to investigate whether clinical effectiveness of controlled dose tapering is non-inferior the usual care with respect to psoriasis activity for patients receiving adalimumab, etanercept or ustekinumab.

### Objectives

The aim of this study is to investigate whether tight controlled dose reduction of biologics in patients with stable plaque psoriasis and low disease activity in combination with good dermatological quality of life is non-inferior compared to therapy with the standard full dose. This will lead us to the following research questions:

### Primary objective

To assess whether a biologic dose tapering strategy in psoriasis patients is non-inferior (NI) to usual care measured by PASI at 12 months with a non-inferiority margin of 0.5.

### Secondary objectives

1. To assess the proportion of patients with successful dose tapering at 12 months. We defined a successful dose tapering as a lower dose than the regular dose and PASI ≤ 5.
2. To compare the quality of life (Dermatology Quality of Life index, DLQI) maintenance between the dose-reduction group and usual care at month 3,6,9 and 12. If the difference at 12 months between the two strategies is smaller than a NI margin of 2 we can consider the strategies non-inferior regarding quality of life.
3. To compare disease activity (PASI) at month 3,6,9 and 12 between the dose reduction group and usual care.
4. To compare the proportion of patients with persistent flares between the dose reduction group and usual care.
5. To identify factors which are associated with successful dose-tapering. >The following factors will be studied: baseline variables such as patient- disease and treatment characteristics, CRP (C- Reactive protein), pharmacogenetic markers (HLA-C*06), anti-drug antibody and trough levels.
6. To compare the occurrence of serious adverse events (SAEs) between dose-reduction and usual care.
7. To calculate decremental cost-effectiveness of the intervention compared to usual care.

## Methods

### Study setting

This study will be carried out in six departments of dermatology in the Netherlands: one academic center (Radboud University Medical Center, Nijmegen) and five non-academic regional centers (St. Anna Hospital in Geldrop, Ziekenhuisgroep Twente Almelo and Hengelo, Gelre Hospital Apeldoorn and Slingeland Hospital Doetinchem. Ethical approval for this study was obtained from the Medical Ethical Committee (Arnhem- Nijmegen). Written informed consent will be obtained from each participant.

### Eligibility

Patients who are diagnosed with plaque psoriasis and have been using etanercept, adalimumab or ustekinumab in a stable dose and have a sustained low disease activity both for at least 6 months are eligible for this study. We defined a sustained low disease activity as a PASI score ≤ 5 for at least 6 months before inclusion (2 PASI scores should available) and a DLQI ≤5 at moment of inclusion. A PASI score ≤ 5 is chosen based on experts opinion and it is in general accepted as low disease activity. A DLQI score gives an indication of what the impact of psoriasis is on the quality of life of these patients [16].

In order to establish whether a good disease control is present, a combination of not only clinical outcome measure (PASI) but also a patient outcome measure (DLQI) is assessed at baseline, month 3,6,9 and 12. Patients who are eligible for this study must meet the following criteria:

### Inclusion criteria

- Age ≥ 18 years.
- Diagnosis of plaque psoriasis established by a dermatologist.
- Sustained low disease activity (PASI ≤ 5) for at least 6 months before inclusion and DLQI ≤ 5 at moment of inclusion.
- Receiving stable and standard dosing treatment with adalimumab, etanercept, or ustekinumab for at least 6 months
- Ability to understand informed consent, read and answer questionnaires.
- All medication except for systemic immunosuppressants other than methotrexate or acitretine is allowed during the study.

### Exclusion criteria

A potential subject who meets any of the following criteria will be excluded from participation in this study:

- Psoriasis not being the main reason for biologic prescription (e.g. when a patient has RA and psoriasis, and RA is the main reason for biologic use).
- Concomitant use of systemic immunosuppressants other than methotrexate or acitretin for psoriasis. Use of corticosteroid inhalations is permitted.
- When the biologic has been stopped for more than 4 weeks in the past 6 months.
- Severe comorbidities with short life-expectancy (e.g. metastasized malignancy).
- Presumed inability to follow the study protocol.

### Design

This is a multicenter, randomized non-inferiority trial with a cost effectiveness analysis. This trial is currently being conducted at the department of dermatology of six medical centers. (see above in Study Setting) in the Netherlands.

In this study a comparison will be made between an intervention group (dose-tapering) and a group receiving usual care. In the intervention arm we will reduce the dose of the biologic by means of prolongation of the intervals between two doses. We aim to determine whether we can achieve the same clinical effect with administration of a reduced dose of the biologic compared to the standard full dose. Consequently, we chose for a randomized controlled non-inferiority trial.

We aim to include patients on either adalimumab, etanercept or ustekinumab to an equal extent. Infliximab was excluded as intermittent therapy increases the risk for infusion reactions. No other biologics had been approved at the moment of designing the study and were therefore not included.

### Recruitment

All patients who are eligible for this study will be asked by their dermatologist and they will receive oral and written information from the local investigator. The investigator will obtain written informed consent and the patient will be randomized. The schedule of their biological will be explained depending on which biologic the patient uses.

Subjects can leave the study at any time for any reason if they wish to do so without any consequences. The investigator can decide to withdraw a subject from the study for urgent medical reasons. When subjects are withdrawn from the study, they will not be replaced.

### Randomization, blinding, treatment allocation

The investigator will generate the allocation sequence, enroll patients and will assign participants to interventions. Randomization of the subjects will be carried out web-based with stratification by agent using variable permuted blocks with concealment of allocation. Patients will be randomized into 1:1 (1) dose- tapering strategy or (2) drug continuation strategy of the standard dose of adalimumab, etanercept or ustekinumab. Every three months patients will be seen at the outpatient clinic and the PASI and DLQI will be performed.

Patients in the dose-tapering group will receive adalimumab, etanercept or ustekinumab, and doses will be lowered according to the schedule as described below. Patients in the non dose-tapering group will receive the standard dose without interval prolonging.

### Study groups

#### Control group

Patients in the control group will continue the treatment with the normal dose and treatment regimens will be based on the prevailing guidelines. Treatment decisions are made at the discretion of the treating dermatologist following these guidelines. Control visits are planned every three months and patients are explained to contact their dermatologist when they experience increased disease activity. The PASI, DLQI questionnaires and CRP are determined when patients visit their dermatologist. The research physician will observe whether the dermatologist follows the study protocol and will give advice when necessary. In case of a disease flare treatment will be adjusted, topical and systemic therapy will be optimized and when required the dose of the biologic will be increased or treatment will be switched to another agent.

#### Dose-tapering group

**Fig. 1:**
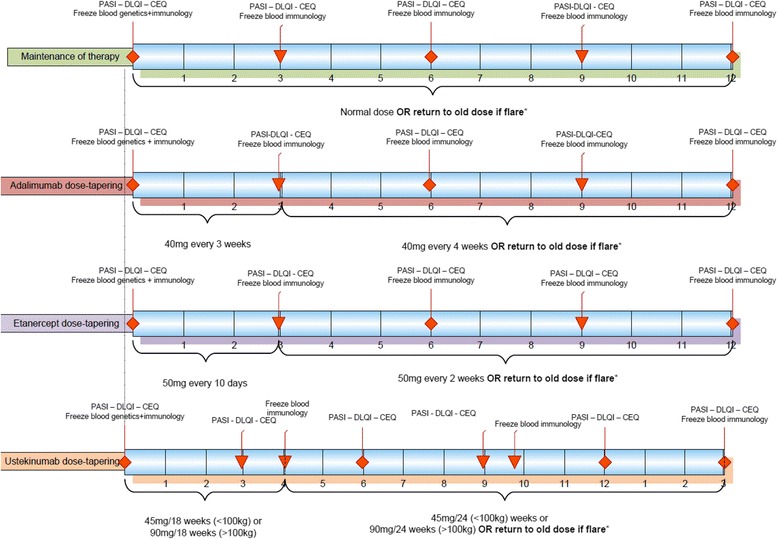
Patients using adalimumab, etanercept or ustekinumab will be randomized to dose tapering or usual care. Control visits will be planned every 3 months for the assessments of PASI, DLQI, cost-effectiveness questionnaires, drug levels and anti-drug antibodies (at trough moment). Ustekinumab blood samples for immunologic analyses are taken at deviating time points (trough moments) due to the low frequency of injections. CEQ = Cost-effectiveness questionnaires (SF-36, iMTA Medical Consumption Questionnaire and productivity cost questionnaire), PASI = Psoriasis Area and Severity Index, DLQI = Dermatology Quality of Life

In the dose tapering group the doses of etanercept, adalimumab or ustekinumab will be lowered according to a predefined schedule. The intervals of drug-administration will be prolonged stepwise depending on the PASI and DLQI score. Dose-tapering is allowed when the PASI score ≤5 and the DLQI score is ≤5. First, the dose will be decreased to 66–70% of the normal dose of the biologic (by interval prolongation with a factor 1.5). After 3 months, if the patients remain in a state of low disease activity, the dose will be further reduced to 50% of the original dose (by doubling the original interval). This method of dose reduction is visualized in Fig. 1. The dose-reduction steps per specific drug are as follows: For etanercept the interval will be prolonged stepwise from 7 days to 10 days to 14 days. For adalimumab, the interval will be prolonged from 14 to 21 to 28 days. For ustekinumab the interval will be stepwise increased from 12 to 18 to 24 weeks. If possible, the patient will stay on the lowest interval. When disease flare occurs, confirmed by the dermatologist or research physician, the patient will return to the previous effective dose interval. When there is still no disease control after reintroduction of the normal dose, treatment will be adjusted: topical and systemic therapy will be optimized and when required the dose of the biologic will be increased or treatment will be switched to another agent.

### Procedures and outcome measures

At baseline the patient and treatment characteristics will be measured and collected, such as sex, age, previous conventional antipsoriatic drugs and/or biologics used, disease duration, PASI and DLQI scores. Baseline measures will also be extracted from the existing BioCAPTURE database and will be used for identifying predictors for successful dose tapering. The BioCapture database is a prospective cohort of patients with psoriasis using biologicals in daily practice [17]. Genetic factors or polymorphisms influencing response on biologicals, such as HLA-C*6, may be related to successful dose-tapering and will also be measured [18].

Blood samples will be drawn for safety, genetic analysis, determining antibodies and trough levels. Patients will be followed for 1 year and regular visits will be planned at baseline, month 3,6,9 and 12. Every visit the PASI and DLQI score, CRP, anti- drug antibody, trough levels, disease flares, costs, health status, questionnaires, Severe Adverse Events (SAE) and their causal relation with the biologic will be determined. Patients with a disease flare will be seen within a week and additional visits or telephone contact will be planned within 2 or 4 weeks.

Our primary outcome measure is disease activity (PASI) at 12 months and will be analyzed with ANCOVA in which the baseline PASI will be included as covariate to gain efficiency. If the lower limit of the 95% confidence interval around the mean difference in PASI score between the intervention group and control group does not exceeds the non- inferiority margin (−0.5) at 12 months of follow up, the intervention can be regarded as non-inferior to usual practice.

Our secondary study outcome measures are as follows:

the DLQI, the number of patients with 1 or more persistent flares (defined as having at least 3 months PASI score >5 or DLQI > 5), treatment characteristics (dose of the biologic, drug pauses, use of concomitant antipsoriatic systemic drugs, use of topical therapies), CRP, anti- drug antibodies and trough levels, SAE and their causal relation with the biologic, costs questionnaires and health status.

### Definition of (persistent) disease flare

A (short) psoriasis flare in this study is defined as a PASI score > 5 and/or DLQI score > 5, a persistent flare is defined as a PASI score > 5 and/or the DLQI score >5 for at least 3 months. DLQI ≤ 5 means mild influence on quality of life and is part of the published treatment goals [6, 19, 20]. Zweegers et al. showed that the mean PASI of patients that remain on a biologic is ≤ 5 [21]. Moreover, PASI ≤ 5 is associated with low DLQI scores [22]. Therefore we think that PASI ≤5 is a good threshold for minimal disease activity in general. In the exceptional case were PASI 5 still represents relatively active disease, DLQI score is expected to be high and patients will still be classified as having a flare.

### Power and sample size analyses

We estimated that per arm 54 patients should be included to provide sufficient power for statistical analyses (P 80%). The non-inferiority (NI) margin of 0.5 was based on our opinion of clinical meaningfulness. Also, as patients in clinical remission in the BioCAPTURE cohort showed a PASI standard deviation (SD) of 1.4, the margin of 0.5 is 36% of the SD. This percentage approximates a rule of thumb for calculating the non-inferiority margin: 1/3 of SD. As we will perform our analysis with correction for the baseline value of PASI, a formula that will increase power significantly is available taking into account the correlation between the baseline value and the follow-up measurements [23]. This correlation was found to be a 0.67 in our existing cohort. When performing a *t*-test for sample size calculation, the sample size can be multiplied by 1- ρ2 if we add one extra subject per group, where ρ is the correlation between the baseline measure and the follow-up measure here (0.67). Thus when we perform an ANCOVA analysis correcting for baseline PASI with the non-inferiority margin set at 0.5, α set at 5% and a ρ of 0.67, we achieve 80% power when enrolling 54 patients per arm. Taking a possible drop-out rate of 10% into account, 60 patients need to be included in each arm.

### Data collection and management

All data will be collected and entered in CASTOR. This is an electronic data management system which is setup for clinical trials [24]. Data will be coded and kept based on the rules for good clinical practice (GCP) by GCP certified personnel [25]. Handling of personal data will comply with the Dutch Personal Data Protection Act [26].

All blood samples will be coded before sending to the dermatology laboratory at the Radboudumc and Sanquin where the samples will be analyzed. Blood for pharmacogenetic investigations will also be coded before work-up in the genetic department of the Radboudumc.

### Statistical analysis

Per protocol analyses will be performed as a primary method for the most outcomes as this is preferred and most conservative analysis for non-inferiority studies [27]. Lost to follow up subjects will be incorporated until their lost to follow up date only, as it is a per protocol approach. However, for the cost-effectiveness analysis an intention- to- treat analysis will be performed separately. Extent and nature of missing data and information about lost to follow up subjects will be described. For the intention-to-treat analysis a lost observation carried forward approach will be used for those that were lost to follow up. In case of missing data for patients that did complete the follow-up imputation of missing data will be performed.

The primary study outcome (PASI at 12 months) will be analyzed with ANCOVA. The baseline PASI will also be included as a covariate to gain efficiency. The proportion of patients with successful dose tapering will be assessed. The DLQI and PASI scores, antidrug antibodies and trough levels will be directly compared at time points month 3,6,9 and 12 between the two groups using an unpaired *t*-test or a non- parametric alternative. The DLQI will be formally tested on non-inferiority. If the upper limit of the 95% confidence interval of the difference between the two strategies lower than the NI margin of 2, we can consider the strategies non-inferior regarding quality of life. This NI margin of 2 is approximately a 1/3 of the standard deviation (data on file) which complies with a general rule of thumb for determining a NI margin. Furthermore, a difference in DLQI larger than 2 can be considered as clinically relevant. The number of persistent flares for both groups will be analyzed as rate ratios and relative ratios with some confidence intervals. With Kaplan-Meier curves the time until flare for dose tapering will be presented. All SAEs will be described per group in a frequency table. Thereafter, cost effectiveness analyses will be calculated based on the questionnaires medical consumption, productivity costs and health status (SF-36). Because we anticipate non-inferiority we will primarily analyze cost-savings: direct medical cost as well as total costs (medical and non-medical costs) will be compared between dose tapering strategy and usual care. A possible small but acceptable loss of effect can be incorporated in the analyses by determining a decremental cost-effectiveness ratio (DCER) by dividing the difference in costs by the difference in Quality Adjusted Life Years (QALYs) between the groups. The DCER expresses with how much money a loss of 1 QALY is compensated. If this amount is high, the decision makers are willing to accept a loss of effect. Furthermore, the Net Monetary Benefit (NMB) per patient will be calculated for different levels of willingness to pay (WTP) in dollars per QALY, using the formula: WTP * effect (difference in QALY) - costs. This results in the net amount of money saved, when the possible loss of QALY is corrected for, using different WTP levels per QALY.

### Immunological analyses

Immunologic analyses will be done for each biologic to determine anti-drug antibodies/trough level and logistic regression analyses will be carried out to identify predictors that are related to the absence of flare. The four most promising variables will be tested.

### Monitoring

Data of all centers will be monitored following the guidelines of the Radboudumc. The research nurse of the Radboudumc will monitor all centers involved in this study.

## Discussion

Without doubt biologics represent a very effective modality to treat patients with moderate to severe psoriasis. Dose-reduction of biologics while maintaining clinical effectiveness is a promising way to improve safety and reduce the costs of this type of therapy. The relatively short follow up time could be considered as a limitation. For this reason we will follow patients during 1 year after the end of this initial study. This will lead to insight in the long-term effects of dose reduction. Successful dose-reduction of adalimumab and etanercept has been demonstrated in patients with RA [15], however there are only limited data for patients with psoriasis. In this prospective randomized non- inferiority study the possibility will be investigated of dose-reduction of the most used biologics for psoriasis: adalimumab, etanercept and ustekinumab. If dose-reduction is non-inferior to the current care with standard dosages, we can treat patients with lower dosages of biologics in daily practice. This can lead to a lower risk of side effects and reduce health care costs.

## Abbreviations

PASI: Psoriasis area and severity index
DLQI: Dermatology quality of life index
NI: Non- inferior
RA: Rheumatoid arthritis
CRP: C-reactive protein
SAE: Serious adverse events
SD: Standard deviation
GCP: Good clinical practice
CEQ: Cost-effectiveness questionnaire

## References

[1] Rapp SR, Feldman SR, Exum ML, Fleischer AB, Reboussin DM (1999). Psoriasis causes as much disability as other major medical diseases. J Am Acad Dermatol.

[2] Driessen RJ, Berends MA, Boezeman JB, van de Kerkhof PC, de Jong EM (2008). Psoriasis treatment with etanercept and efalizumab: clinical strategies influencing treatment outcome. Br J Dermatol.

[3] Leonardi CL, Kimball AB, Papp KA, Yeilding N, Guzzo C, Wang Y, Li S, Dooley LT, Gordon KB (2008). Efficacy and safety of ustekinumab, a human interleukin-12/23 monoclonal antibody, in patients with psoriasis: 76-week results from a randomised, double-blind, placebo-controlled trial (PHOENIX 1). Lancet.

[4] Gordon KB, Gottlieb AB, Langely RG, van de Kerkhof P, Belasco KT, Sundaram M, Okun M, Serra L (2015). Adalimumab retreatment successfully restores clinical response and health-related quality of life in patients with moderate to severe psoriasis who undergo therapy interruption. J Eur Acad Dermatol Venereol.

[5] Welsing PM, Bijl M, van Bodegraven AA, Lems WF, Prens E, Bijlsma JW (2011). Cost effectiveness of biologicals: high costs are the other face of success. Ned Tijdschr Geneeskd.

[6] Mrowietz U, de Jong EM, Kragballe K, Langley R, Nast A, Puig L, Reich K, Schmitt J, Warren RB (2014). A consensus report on appropriate treatment optimization and transitioning in the management of moderate-to-severe plaque psoriasis. J Eur Acad Dermatol Venereol.

[7] Menter A, Tyring SK, Gordon K, Kimball AB, Leonardi CL, Langley RG, Strober BE, Kaul M, Gu Y, Okun M (2008). Adalimumab therapy for moderate to severe psoriasis: a randomized, controlled phase III trial. J Am Acad Dermatol.

[8] Papp K, Crowley J, Ortonne JP, Leu J, Okun M, Gupta SR, Gu Y, Langley RG (2011). Adalimumab for moderate to severe chronic plaque psoriasis: efficacy and safety of retreatment and disease recurrence following withdrawal from therapy. Br J Dermatol.

[9] Gordon KB, Gottlieb AB, Leonardi CL, Elewski BE, Wang A, Jahreis A, Zitnik R (2006). Clinical response in psoriasis patients discontinued from and then reinitiated on etanercept therapy. J Dermatolog Treat.

[10] Moore A, Gordon KB, Kang S, Gottlieb A, Freundlich B, Xia HA, Stevens SR (2007). A randomized, open-label trial of continuous versus interrupted etanercept therapy in the treatment of psoriasis. J Am Acad Dermatol.

[11] Blum R, Lebwohl M (2005). Durability of treatment response in patients with moderate to severe psoriasis following withdrawal from or a dose reduction in adalimumab therapy. J Am Acad Dermatol.

[12] Papp K, Menter A, Poulin Y, Gu Y, Sasso EH (2013). Long-term outcomes of interruption and retreatment vs. continuous therapy with adalimumab for psoriasis: subanalysis of REVEAL and the open-label extension study. J Eur Acad Dermatol Venereol.

[13] Na JI, Kim JH, Park KC, Youn SW (2008). Low-dose etanercept therapy in moderate to severe psoriasis in Korean. J Dermatol.

[14] Rodrigo- Nicolas B G-CE. Adalimumab dose reduction in psoriasis: Results in a series of 12 patients. J Am Acad Dermatol. 2014:7775

[15] van Herwaarden N, van der Maas A, Minten MJ, van den Hoogen FH, Kievit W, van Vollenhoven RF, Bijlsma JW, van den Bemt BJ, den Broeder AA (2015). Disease activity guided dose reduction and withdrawal of adalimumab or etanercept compared with usual care in rheumatoid arthritis: open label, randomised controlled, non-inferiority trial. BMJ.

[16] Finlay AY, Khan GK (1994). Dermatology life quality index (DLQI)--a simple practical measure for routine clinical use. Clin Exp Dermatol.

[17] Zweegers J, Groenewoud JM, van den Reek JM, Otero ME, van de Kerkhof PC, Driessen RJ, van Lumig PP, Njoo MD, Ossenkoppele PM, Mommers JM et al. Comparison of the one and 5-years effectiveness of adalimumab, etanercept and ustekinumab in psoriasis patients in daily clinical practice: Results from the prospective BioCAPTURE registry. Br J Dermatol. 2016;176(4):1001–1009. 10.1111/bjd.15023 27579864

[18] Talamonti M, Botti E, Galluzzo M, Teoli M, Spallone G, Bavetta M, Chimenti S, Costanzo A (2013). Pharmacogenetics of psoriasis: HLA-Cw6 but not LCE3B/3C deletion nor TNFAIP3 polymorphism predisposes to clinical response to interleukin 12/23 blocker ustekinumab. Br J Dermatol.

[19] Hongbo Y, Thomas CL, Harrison MA, Salek MS, Finlay AY (2005). Translating the science of quality of life into practice: what do dermatology life quality index scores mean?. J Investig Dermatol.

[20] Mrowietz U, Kragballe K, Reich K, Spuls P, Griffiths CE, Nast A, Franke J, Antoniou C, Arenberger P, Balieva F (2011). Definition of treatment goals for moderate to severe psoriasis: a European consensus. Arch Dermatol Res.

[21] Zweegers J, Roosenboom B, van de Kerkhof PC, van den Reek JM, Otero ME, Atalay S, Kuijpers AL, Koetsier MI, Arnold WP, Berends MA et al.: Frequency and predictors of a high clinical response in patients with psoriasis on biological therapy in daily practice: results from the prospective, multicenter BioCAPTURE cohort. Br J Dermatol. 2016 10.1111/bjd.14888 27454758

[22] Reich K, Nestle FO, Papp K, Ortonne JP, Wu Y, Bala M, Evans R, Guzzo C, Li S, Dooley LT (2006). Improvement in quality of life with infliximab induction and maintenance therapy in patients with moderate-to-severe psoriasis: a randomized controlled trial. Br J Dermatol.

[23] Borm GF, Fransen J, Lemmens WA (2007). A simple sample size formula for analysis of covariance in randomized clinical trials. J Clin Epidemiol.

[24] https://www.castoredc.com/. Accessed 15 Nov 2016.

[25] GCP good clinical practice hin, http://www.federa.org/code-goed-gedrag coc. Accessed 15 Nov 2016.

[26] WBP, wet bescherming persoonsgegevens. https://autoriteitpersoonsgegevens.nl/nl/over-privacy/wetten/wet-bescherming-persoonsgegevens.

[27] Soonawala D, Dekkers OM (2012). ‘Non-inferiority’ trials. Tips for the critical reader. Research methodology 3. Ned Tijdschr Geneeskd.

